# Haematopoietic cell‐derived exosomes in cancer development and therapeutics: From basic science to clinical practice

**DOI:** 10.1002/ctm2.1448

**Published:** 2023-10-13

**Authors:** Wen‐Chun Lin, You‐Tong Lin, Hui‐Ching Chao, Yen‐Yu Lin, Wei‐Lun Hwang

**Affiliations:** ^1^ Department of Biotechnology and Laboratory Science in Medicine National Yang Ming Chiao Tung University Taipei Taiwan; ^2^ Department of Pathology, Fu Jen Catholic University Hospital Fu Jen Catholic University New Taipei City Taiwan; ^3^ School of Medicine, College of Medicine Fu Jen Catholic University New Taipei City Taiwan; ^4^ Cancer and Immunology Research Center National Yang Ming Chiao Tung University Taipei Taiwan

**Keywords:** blood exosomes, cancer therapeutics, exosome engineering, tumour microenvironment

## Abstract

**Background:**

The tumour microenvironment (TME) is a specialised niche involving intercellular communication among cancer cells and various host cells. Among the host cells, the quantity and quality of immune cells within the TME play essential roles in cancer development and management. The immunologically suppressive, so‐called ‘cold’ TME established by a series of tumour–host interactions, including generating immunosuppressive cytokines and recruiting regulatory host immune cells, is associated with resistance to therapies and worse clinical outcomes.

**Main body:**

Various therapeutic approaches have been used to target the cold TME, including immune checkpoint blockade therapy and adoptive T‐cell transfer. A promising, less explored therapeutic strategy involves targeting TME‐associated exosomes. Exosomes are nanometer‐sized, extracellular vesicles that transfer material from donor to recipient cells. These particles can reprogram the recipient cells and modulate the TME. In particular, exosomes from haematopoietic cells are known to promote or suppress cancer progression under specific conditions. Understanding the effects of haematopoietic cell‐secreted exosomes may foster the development of therapeutic exosomes (tExos) for personalised cancer treatment. However, the development of exosome‐based therapies has unique challenges, including scalable production, purification, storage and delivery of exosomes and controlling batch variations. Clinical trials are being conducted to verify the safety, feasibility, availability and efficacy of tExos.

**Conclusion:**

This review summarises our understanding of how haematopoietic cell‐secreted exosomes regulate the TME and antitumour immunity and highlights present challenges and solutions for haematopoietic cell‐derived exosome‐based therapies.

## Graphical abstract

Haematopoietic cell‐derived exosomes exert tumour‐promoting and inhibitory effects in the tumour microenvironment. Therapeutic exosomes are modified to enhance stability, specificity and drug‐carrying capacity for combinatorial therapy. Achieving scalable production and quality control during exosome manufacturing is challenging for personalised medicine.

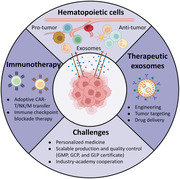

## 1 INTRODUCTION

Blood comprises red blood cells (RBCs), T cells, B cells, natural killer (NK) cells, granulocytes, monocytes and platelets that developed and matured from stem/progenitor cells in defined niches.^1^ Cell‐free components include blood gases, electrolytes, nucleic acids, proteins and metabolites. Circulating blood delivers nutrients to organs and removes cellular waste to maintain homeostasis under physiological conditions. In tissues, monocytes can differentiate into macrophages or dendritic cells (DCs) in response to local stimuli.^2^ Changes in blood components indicate an organism's status. Blood parameters, such as complete blood counts, differential cell counts, blood pH, enzymatic activities or tumour markers, can be used for disease diagnosis, tracking patient status and therapeutic interventions in clinical practice. Plasma and serum specimens have been considered sources of liquid biopsy, offering a manageable method for clinical manifestation and biomarker discovery.^3^ Extracellular vesicles (EVs) mediate regional and distal communication. For years, EVs have garnered attention as biomarkers in various body fluids due to the abundance of EVs and EV‐encapsulated nucleic acids, proteins, lipids and metabolites in specific diseases.^4^, ^5^ Tumour microenvironment (TME) orchestration plays an essential role in oncogenesis. Accumulating evidence shows that the release of exosomes, a type of endocytic, nanoscale EV, from tumour and host immune cells participates in cancer initiation, progression and antitumour efficacy.^6^, ^7^ With advances in chimeric antigen receptor (CAR)‐T/NK‐cell therapy, advantages of CAR‐macrophages (CAR‐M) in cancer treatment^8^ and the demonstrations of therapeutic exosomes (tExos) from mesenchymal stem cells (MSCs) in treating human disease,^9^ the scalable production of tExos during cell therapy preparation holds promise for personalised cancer treatment. This review provides a comprehensive discussion on the molecules in the blood cell‐derived exosomes that affect cancer progression and antitumour immunity and emphasises the clinical use of tExos in therapeutics.

### 1.1 The discovery of extracellular vesicles

**FIGURE 1:**
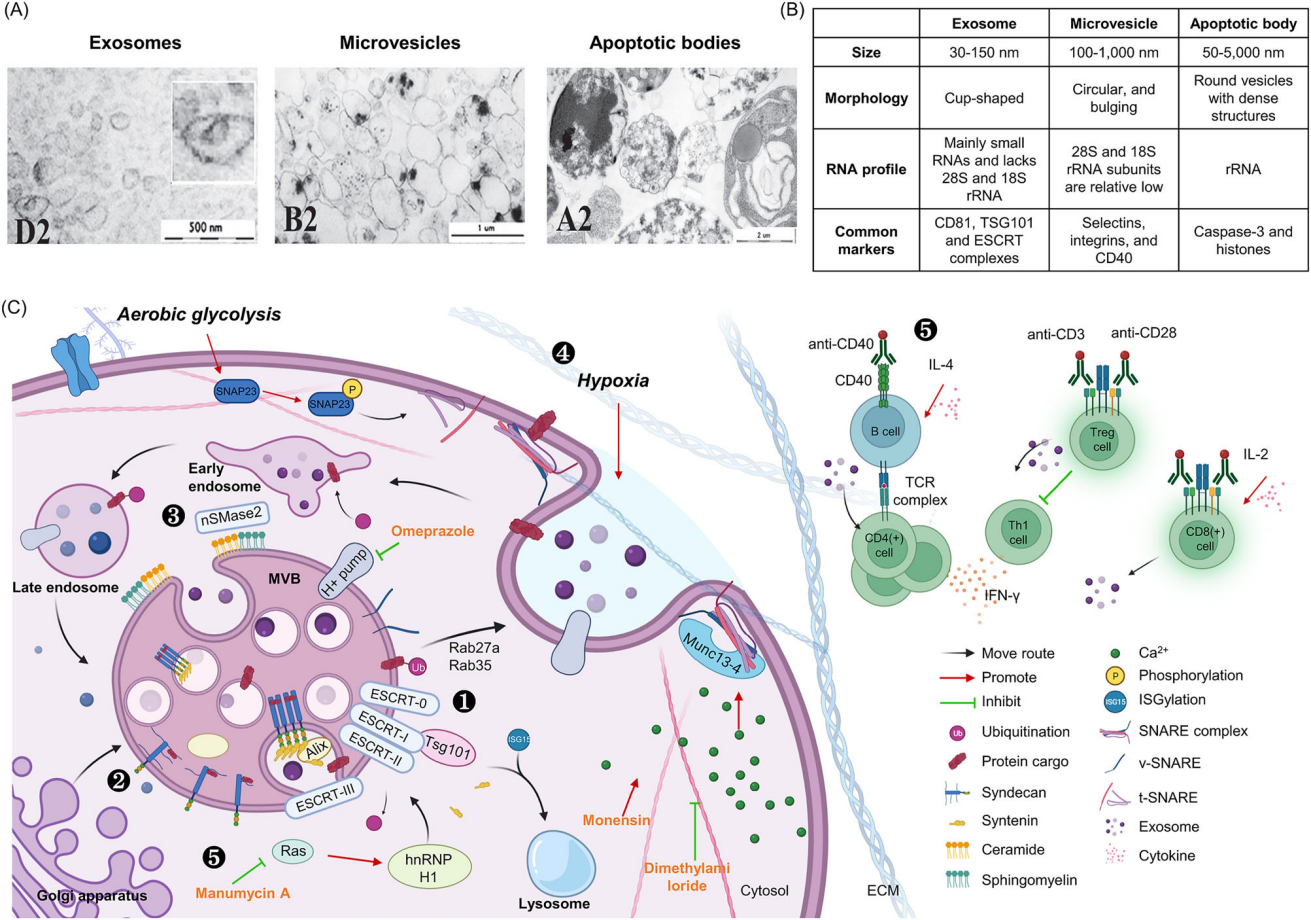
Exosome features and molecular mechanisms that regulate exosome secretion. (A) Images of exosomes, microvesicles and apoptotic bodies from human erythroleukaemia cell TF‐1 by transmission electron microscopy (TEM). The images are reproduced with permission from reference.^16^ (B) A table summarises the features of indicated extracellular vesicles. (C) The image shows how cells use different pathways to generate exosomes and modulate exosome secretion: (1) in the endosomal sorting complexes required for the transport (ESCRT) pathway, ubiquitinylated proteins are recruited by ESCRT‐0 and loaded into intraluminal vesicles (ILVs) after ubiquitin removal. (2) Certain cargo linked to syndecans undergoes heparanase modification and is associated with syntenin for ILV entry through Alix. (3) The ceramide pathway involves neutral sphingomyelinase (nSMase) converting sphingomyelin to ceramide to induce inward membrane curvature to facilitate cargo entry into ILVs. (4) Increased exosome secretion can be observed under aerobic glycolysis, tumour hypoxia, T‐cell receptor (TCR) engagement and proinflammatory conditions. (5) Specific small compounds are also used to modulate the exosome secretion. Manumycin A inhibits Ras to inhibit hnRNP H1 transcription, thereby affecting cargo sorting. Monensin increases Ca^2+^ concentration by enhancing Na^+^ influx. Dimethyl amiloride inhibits H^+^/Na^+^ and Na^+^/Ca^2+^ exchangers, thereby suppressing the increase in Ca^2+^ concentrations. Omeprazole affects pH levels by inhibiting H^+^ pumps, subsequently influencing exosome secretion. The figure was created using BioRender.

The first research on blood EVs can be traced back to 1899 when Edward G. Horder observed granules secreted from white blood cells (WBCs).^10^ In 1967, Wolf obtained a lipid‐rich substance secreted during platelet activation called platelet dust.^11^ In 1987, Johnstone et al. isolated sphingomyelin‐rich vesicles from sheep reticulocytes.^12^ Research on non‐blood cell EVs dates back to 1966 when Sun observed dense laminated structures of different sizes and shapes derived from alveolar cells in rat lungs.^13^ Next, Bonucci found the presence of periodic acid‐Schiff‐reactive and osmiophilic extracellular nanovesicles with small needle‐shaped apatite crystallites near the calcification area in cartilage tissues.^14^ As research has progressed, EVs have been mainly grouped as exosomes, microvesicles (MVs) and apoptotic bodies.^15^, ^16^ Exosomes generate endocytic pathways with a diameter of approximately 30−150 nm and exhibit a cup‐shaped morphology under transmission electron microscopy. Proteins related to vesicle trafficking, such as tetraspanins (CD81) and members of the endosomal sorting complexes required for the transport (ESCRT) complexes (TSG101), are predominant in exosomes. Exosomal RNAs are mainly small RNAs and lack ribosomal RNA (rRNA) subunits 28S and 18S. MVs are mainly produced through the budding of the cell membrane and are larger in size, ranging from 100 to 1000 nm, and show circular and bulging structures. MVs are characterised by the expression of selectins, integrins and CD40. The 28S and 18S rRNA subunits are low or lacking in MVs. Apoptotic bodies generated during programmed cell death have a broad range of sizes, varying from 50 to 5000 nm in diameter, and exhibit round morphology with dense chromatin substance. Caspase‐3 and histones are common markers associated with apoptotic bodies. RNA profiling shows abundant rRNAs in apoptotic bodies. The vesicular features and images of EVs are summarised in Figure 1A,B.

### 1.2 Mechanisms that regulate exosome biogenesis

ESCRT is a conserved pathway for exosome biogenesis and includes the ESCRT‐0, ESCRT‐I, ESCRT‐II and ESCRT‐III complexes. ESCRT‐0 recognises ubiquitinated proteins in endosomal membranes. ESCRT‐I and ESCRT‐II limit membranes into the multivesicular body (MVB) lumen. ESCRT‐III assembles into circular arrays that constrict the budding necks. Vps4 facilitates membrane abscission, leading to the formation of intraluminal vesicles (ILVs) within MVBs. These ILVs are released as exosomes when MVBs fuse with the plasma membrane.^17^ The ESCRT accessory component protein Alix interacts with ESCRT‐I by binding with TSG101 and participates in the budding and abscission processes.^18^ The syndecan–syntenin–Alix pathway regulates ILV formation. Syndecan supplies most cell‐bound heparan sulphate, thereby facilitating cell surface signalling. Syndecan is connected with Alix through the cytoplasmic adaptor, syntenin, which promotes ILV biogenesis and the confinement of signalling molecules to these vesicles.^19^ Heparanase further stimulates the formation of syntenin‐1‐containing exosomes through the syndecan–syntenin–Alix pathway by trimming heparan sulphate chains on syndecans.^20^ Additionally, the neutral sphingomyelinase induces ILV formation and exosome release by increasing sphingolipid ceramide production from sphingomyelin.^21^ The Rab family of small GTPases also participates in exosome secretion. Rab27A regulates the docking and membrane fusion of MVBs, while Rab27B modulates membrane transfer from the trans‐Golgi network to MVBs in HeLa cells.^22^ In Oli‐neu oligodendroglial cells, Rab35 regulates exosome secretion.^23^ Manumycin A inhibits Ras activation and suppresses the expression of Alix and Rab27A, leading to the attenuation of exosome secretion in castration‐resistant prostate cancer.^24^

Perturbations can modulate exosome secretion. Hypoxic conditions enhance exosome release in human breast cancer cells.^25^ With Ca^2+^, the Ca^2+^‐dependent SNAP receptor and Rab‐binding protein Munc13‐4 interact with active Rab11a to fuse Rab11(+) endosomes with MVBs, ultimately facilitating exosome secretion by MDA‐MB‐231 cells.^26^ Monensin increases intracellular Ca^2+^, thereby enhancing the exosome release in K562 cells.^27^ Modulating cellular calcium levels (dimethyl amiloride) or pH regulation via proton pumps (omeprazole) can also influence exosome release.^28^

Posttranslational modifications determine MVB fates. ISG15‐mediated ISGylation of TSG101 promotes the fusion of MVBs with lysosomes, which then undergo degradation in HEK293 cells.^29^ In A549 cells, phosphorylated pyruvate kinase M2 promotes the phosphorylation of SNAP23, thereby facilitating SNARE complex formation and exosome release.^30^ These cancer‐derived exosomes can inactivate host immunity. Cancer exosomal CD73 and CD39 promote the hydrolysis of 5′adenosine monophosphate and adenosine triphosphate (ATP) to adenosine to suppress T‐cell immune activity.^31^ Exosomal programmed death‐ligand 1 (PD‐L1) also contributes to immune suppression. In metastatic melanomas, interferon‐gamma (IFN‐γ) has been shown to increase surface PD‐L1 expression on exosomes. This upregulation of PD‐L1 hinders the effectiveness of PD‐L1 antibodies, suppresses CD8(+) T cells, and leads to immune evasion.^32^

### 1.3 Exosome secretion by haematopoietic cells

Changes in exosomal composition and quantity change with the development of blood cells. Maturing erythroid cells undergo the transferrin receptor (TfR)‐mediated endocytosis to internalise iron‐bound transferrin for hemoglobin synthesis. During erythropoiesis, proteins related to ion channels and transporters are first loaded onto exosomes, and an increase in exosomal ESCRT proteins was observed later.^33^ An increase in exosomal TfR was noted during erythrocyte maturation,^34^, ^35^ indicating a systematic process of exosome release.

Human CD4(+) T cells, CD8(+) T cells and Jurkat leukaemia cells showed enhanced secretion of exosomes carrying the T‐cell receptor (TCR)/CD3 complex and cell adhesion‐related molecules such as CD2 and CD18 in response to TCR conjugation. In contrast, phorbol 12‐myristate 13‐acetate and ionomycin‐treated CD4(+) T cells did not significantly change exosome secretion.^36^ The heterogeneous features of exosomes derived from p53‐specific CD4(+) murine T cells were noted. In the presence of TCRs triggered by anti‐CD3 ligation and anti‐CD28 costimulation, exosomes with highly reduced wide‐angle forward scatter (FSC) levels and high fluorescence of the lipophilic dye PKH‐67 (FSC^high^FL^high^) were observed compared to other populations, suggesting that T‐cell activation shapes pooled exosome features.^37^ Anti‐CD3 and anti‐CD28 activated regulatory T cells also showed enhanced exosome secretion, and exosomal Let‐7d inhibited Cox‐2 and IFN‐γ production in T helper 1 (Th1) cells.^38^ In primary B cells, costimulation of CD40 and interleukin‐4 (IL‐4) receptors but not lipopolysaccharide (LPS) or dextran enhanced B‐cell proliferation and exosomal secretion, which was accompanied by high levels of major histocompatibility complex (MHC) class I, MHC class II and CD45RA and CD19, suggesting that exosome secretion is not constitutively active in B cells.^39^ After recognising antigen‐specific murine splenic B cells by CD4(+) T cells, CD4(+) T cells further stimulate B cells to produce MHC‐II‐containing exosomes for interactions with TCRs. B‐cell‐released exosomes then promote CD4(+) T‐cell proliferation and differentiation.^40^ Immune cell exosomes could also be used as biomarkers for disease development. Patients with chronic hepatitis C have increased levels of T‐cell‐derived exosomes (T‐Exos). Patients with nonalcoholic liver disease or nonalcoholic steatohepatitis have increased levels of exosomes derived from invariant NK T cells and CD14(+) monocytes.^41^ We summarise exosome biogenesis regulators in haematopoietic and non‐blood cells in Figure 1C.

## 2 LYMPHOID CELL‐DERIVED EXOSOMES

The gold standard for cancer immunity starts with DCs, an antigen‐presenting cell that presents antigens to activate naïve T cells to form mature T cells and trigger an adaptive immune response against tumours. Lymphoid lineage cells, including T, B and NK cells, differentiate from haematopoietic stem cells in response to varieties of haematopoietic cytokine stimulation.^42^ The main T‐cell subtypes are CD4(+) T cells and CD8(+) T cells. CD4(+) T cells, which are Th cells, account for approximately 15% of WBCs in normal peripheral blood (PB). CD4(+) T cells secrete cytokines that induce CD8(+) T‐cell proliferation and promote B‐cell antibody production. CD8(+) T cells, which are cytotoxic T cells, then recognise tumour antigens through MHC‐I and secrete perforin, granzymes and granulysin to kill cancer cells. Aside from the antibody‐dependent cell‐mediated cytotoxicity pathway, the expression of activating‐inhibiting ligands of natural cytotoxicity receptors governs the cytotoxicity of NK cells. IFN‐r derived from NK cells further sustains T‐cell immunity.^43^

### 2.1 Roles of T‐cell‐derived exosomes in cancers

With T cells' prominent antitumour immune response, T‐cell‐derived exosomes may help kill cancer. Exosomal miR‐765 from CD45RO(–)CD8(+) T cells suppressed endometrial cancer progression via the miR‐765/PLP2/Notch pathway. The epithelial–mesenchymal transition, which was enhanced by PLP2 via Notch signalling activation, was inhibited by PLP2 targeting miR‐765. The treatment of CD45RO(–)CD8(+) T‐cell‐derived exosomes prolonged the survival of tumour‐bearing mice.^44^ In addition to cytotoxicity against tumour cells, tumour‐specific CD8(+) T‐cell‐derived exosomes inhibited tumour progression by depleting mesenchymal tumour stromal cells.^45^ Vδ2(+)‐T cells are a dominant population among Vδ‐T cells in human PB and can be activated by antigens without DC presentation. Allogenic Vδ2(+)‐T cells are used for therapy. Wang et al. showed that the allogenic Vδ2(+)‐T‐cell‐derived exosomes induced more apoptosis in Epstein–Barr virus (EBV)‐associated tumour cells via the Fas/FasL and tumour necrosis factor‐related apoptosis‐inducing ligand (TRAIL) pathways and promoted stronger CD4(+) and CD8(+) T‐cell‐mediated antitumour in tumour‐bearing mice than autologous exosomes.^46^

Given the importance of the antitumour activity of lymphocytes, these cells are used for cell therapies. CARs, which are synthetic molecules such as antibodies, are expressed on the surface of immune cells to enhance their ability to recognise tumour‐associated antigens. FDA‐approved CAR‐T cell (CAR‐T) therapies exhibit therapeutic efficacy against leukaemia.^47^ However, cytokine release syndrome (CRS) may lead to hypotension, fever, headache, nausea, neurotoxicity and organ failure because of the excessive cytokine levels.^48^ Conversely, CAR‐NK has less toxicity and less cytokine secretion. Thus, CAR‐NK was considered an alternative.^49^

Immune cell‐derived exosomes are potential therapeutic agents for cancer treatment. Anti‐MSLN CAR‐T‐cell‐derived exosomes (CAR‐T‐Exos) expressed anti‐MSLN on the exosome surface and showed cytolytic activity against mesothelin‐positive triple‐negative breast cancer (TNBC) via perforin and granzyme B.^50^ Critically, no CRS was observed even at the highest dose tested in the preclinical study, indicating the safety of CAR‐T‐Exos.^51^

Despite the antitumoural effect of T‐Exos, it was also found that T‐Exos could promote tumour invasiveness. Cai et al. showed that ovalbumin (OVA)‐activated CD8(+) T‐cell‐derived exosomes promoted the invasion of murine melanoma cells and Lewis lung cancer cells by increasing matrix metalloproteinase (MMP)‐9 expression via Fas signalling.^52^ Wang et al. showed that in hepatocellular carcinoma, exosomes from exhausted CD8(+) T cells with programmed cell death 1 and T‐cell immunoglobulin and mucin domain‐containing protein 3 positivity were taken up by other non‐exhausted CD8(+) T cells, and impaired their function by reducing IFN‐γ and IL‐2 expression.^53^ A study also showed that OVA‐specific CD4(+) T‐cell‐Exos from OTII mice inhibited OVA‐specific DC‐induced CD8(+) T‐cell cytotoxicity to melanoma cells.^54^ Thus, mechanisms explaining the protumoural activity of the T‐Exos derived from antigen‐specific T cells may help optimise the development of tExos.

### 2.2 Protumoural B‐cell‐derived exosomes in cancer

Most B‐cell‐derived exosomes are protumoural. Klinker et al. established human lymphoblastoid cell lines by transforming PB cells with attenuated B95‐8 clones of EBV. The researchers found that FasL was predominantly expressed on B‐cell exosomes but not on the cell surface. These secreted MHC‐II(+)FasL(+) exosomes promoted antigen (TT peptide)‐specific apoptosis in autologous CD4(+) T cells.^55^ This immunosuppressive property is a disadvantage in antitumour but can be a potential immunotherapy for transplantation. Exosomes secreted by B cells inhibit the proliferation of CD8(+) T cells. Research has revealed that B cells from tumour‐bearing mice express increased levels of hypoxia‐inducible factor 1‐alpha (HIF‐1α) protein. HIF‐1α can promote the transcription of RAB27A, thereby increasing CD19(+) exosomes. Moreover, chemotherapy drug‐treated cancer cells release large amounts of ATP, which can be hydrolysed into adenosine by CD39 and CD73 on CD19(+) exosomes, thereby attenuating the activity of CD8(+) T cells. A lower level of serum CD19(+) exosomes correlated with a longer progression‐free survival (PFS) period.^56^

### 2.3 NK‐cell‐derived exosomes

In contrast to B‐cell‐derived exosomes, NK‐cell‐derived exosomes (NK‐Exos) exert antitumour effects. Exosomes from the NK‐cell‐enriched lymphocytes (NKLs) induced the cytotoxicity in various cancer cell lines (HepG2, SW620, MKN74, MCF7 and T98G) in vitro and suppressed tumours in mice bearing MCF7‐derived tumours. Expressions of death receptors (Fas, TRAILR1 and TRAILR2), death ligands (Fas ligand and TRAIL), NK‐activating receptors, NK cytotoxicity receptors and cytokines may account for the antitumour potential of NKL‐derived exosomes.^57^ Wu et al. expanded NK cells and isolated NK‐Exos loaded with cytotoxic proteins, including perforin, granzyme A, granzyme B, granulysin and FasL, and found that NK‐Exos triggered cytochrome C release from mitochondria and ER stress in recipient neuroblastoma (NB) cells.^58^ One study revealed that NK cells pre‐exposed to NB cells could produce more exosomes to educate other NK cells and exert greater cytotoxicity against NB cells through increasing NK cell receptors.^59^ Exosomal miR‐3607‐3p from NK cells also prevented the invasiveness of pancreatic cancer cells by targeting IL‐26.^60^ The tumour suppressor miR‐186 in NK‐Exos exerted cytotoxicity against MYCN‐amplified NB cells. The expression of MYCN was inhibited by exosomal miR‐186. miR‐186 can be delivered via anti‐CD56‐coated liposomal nanoparticles to target CD56 on NB cells or NK cells, thereby suppressing MYCN‐induced NB progression or preventing transforming growth factor beta 1 (TGFβ1)‐mediated NK cell inhibition, respectively, and optimising immunotherapy.^61^

The NK‐92 cell line is commonly used as a model for oncoimmunotherapy and used in clinical trials. NK‐92MI cells are NK‐92 cell derivatives with constitutive IL‐2 expression. NK‐92MI‐derived exosomes (NK92MI‐Exos) expressed FasL and perforin to induce apoptosis in B16F10 melanoma cells. The cytotoxic effects of NK92MI‐Exos against SNU484 gastric carcinoma and HCT‐15 colon cancer cells were observed. Moreover, NK92MI‐Exos did not exert significant cytotoxicity against the normal human kidney cell line Phoenix‐Ampho after 24 h of co‐culture. Compared to the NK cells isolated from peripheral blood mononuclear cells (PBMCs), NK‐92MI cells would be a more feasible NK‐Exo source for convenient and scalable production.^62^ The levels of granzyme A and FasL in NK‐92‐derived exosomes (NK92‐Exos) were higher than those in NK‐Exos obtained from ex vivo expansion^58^ In the presence of IL‐15 and IL‐21 stimulation, NK92‐Exos showed enhanced cytotoxic effects against tumour cells through the upregulation of CD226 (DNAM‐1).^63^

Aside from the tumour‐killing capacities of NK‐Exos, NK‐Exos are often used in drug delivery for combinatorial therapy. Han et al. entrapped paclitaxel (PTX) in NK92‐Exos by electroporation and prepared PTX‐loaded exosomes (PTX‐NK‐Exos). Compared with free PTX, the PTX‐NK‐Exos robustly inhibited tumour growth and induced apoptosis in MCF7 cells at the same dose by enhancing the internalisation of PTX.^64^ The NK‐Exos prepared from NK‐graphene oxide chips and ExoBead‐based isolation showed robust efficiency and purity and exhibited higher cytotoxic effects against circulating tumour cells (CTCs).^65^ Table 1 shows the roles of lymphoid exosomes in TME modulation.

**TABLE 1:** Functions of lymphoid cell‐derived exosomes in the tumour microenvironment.

Donor cells	Exosome purification	Verified exosome markers	Recipient cells	Disease status	Molecules of actions	Reference
Murine Foxp3(+) Treg cells	Ultracentrifugation	CD9(+), CD63(+) and CD81(+)	Murine CD4(+) Th1 cells	Anti‐inflammation	Treg suppresses Th1 via Treg‐exosomal miRNA let‐7d transferring by targeting *Ptgs2* (Cox‐2)	^38^
Human CD45RO(–) CD8(+) T cells	Ultracentrifugation	CD63(+), TSG101(+) and GRP78(–)	Endometrial cancer cell lines (Ishikawa and KLE)	Tumour inhibition	CD45RO(–)CD8(+) T‐cell‐derived exosomal miR‐765 suppress cancer cell development through miR‐765/PLP2/Notch axis regulation	^44^
Murine CD8(+) T cells	Ultracentrifugation	CD9(+), CD63(+), TSG101(+) and ALIX(+)	MSCs	Tumour inhibition	CD8(+) T‐cell exosomes containing cytotoxic miRNA miR‐298‐5p deplete lesional MSCs to inhibit cancer cells from tumour progression and metastasis	^45^
Human Vδ2 T cells	Ultracentrifugation	CD63(+), TSG101(+), CD81(+), ALIX (+) and GRP94(–)	EBV(+) cell lines (SNU‐719 and EBV‐LCL)	Tumour inhibition	Vδ2‐T‐derived exosomes induce the apoptosis of EBV‐associated tumour cell lines through death receptor ligands and stimulate IFN‐γ production from CD4(+) and CD8(+) T cells	^46^
Human CAR‐T cells	Ultracentrifugation	CD63(+)	Breast cancer cell lines (MDA‐MB‐231 and BT‐549)	Tumour inhibition	CAR‐T‐derived exosomes present cytolytic activity against breast cancer cells via perforin and granzyme B	^50^
Human CAR‐T cells	Ultracentrifugation	CD63(+), ALIX (+) and TSG101(+)	EGFR(+) or HER2(+) human cancer cell lines and patient‐derived xenografts	Tumour inhibition	CAR‐T‐derived exosomes kill cancer cells with cytolytic perforin and granzyme	^51^
Murine OT‐I CD8(+) T cells	Ultracentrifugation	CD9(+), HSP70(+), TSG101(+) and GRP94(–)	Murine cancer cell lines (B16 and 3LL)	Tumour progression	CD8(+) T‐cell‐derived exosomes promote melanoma and lung cancer cell metastasis by increasing the expression of MMP‐9 via Fas signalling	^52^
Exhausted CD8(+) T cells from liver cancer patients	Ultracentrifugation	CD63(+)	Non‐exhausted CD8(+) T cells	Tumour progression	Exhausted CD8(+) T‐cell‐derived exosome containing lncRNAs uptaken by non‐exhausted CD8(+) T cells to impair their function	^53^
Murine OT‐II CD4(+) T cells	Ultracentrifugation	LAMP‐1(+)	Murine DCs or CD8(+) T cells	Tumour progression	Antigen‐specific CD4(+) T‐cell‐derived exosomes inhibit DC‐mediated T‐cell stimulation and inhibit antigen‐specific CD8(+) CTL response	^54^
Human B‐cell‐derived lymphoblastoid cells and transformed B cells	Ultracentrifugation	NA	Human CD4(+) T cells	Tumour progression	B‐cell‐derived exosomes expressing FasL and MHC‐II induce the apoptosis of CD4(+) T cells	^55^
Murine splenocytes	Magnetic purification	CD9(+) and CD63(+)	Murine cancer cell lines (B16F10, MC38 and 4T1)	Tumour progression	CD19(+) exosomes inhibit the chemotherapy effect against tumours by hydrolysing ATP through CD39 and CD73 in tumour cells and inhibit CD8(+) T‐cell activity	^56^
Human NKL	Size exclusion using 100 kDa MW cut‐off filter membrane followed by ultracentrifugation	CD63(+)	Human cancer cell lines (HepG2, SW‐620, MKN‐74, MCF‐7 and T98G)	Tumour inhibition	NKL‐cell‐derived exosomes express antitumour effectors for tumour inhibition	^57^
Human NK cells	PEG‐based precipitation	NA	Human cancer cell lines (SupB15 and CHLA255)	Tumour inhibition	NK‐cell‐derived exosomes express cytotoxic proteins to kill cancer cells	^58^
Human NK cells	Ultracentrifugation	ALIX(+) and TSG101(+)	NB cell lines (SK‐N‐SH and CHLA‐255) and human NK cells	Tumour inhibition	NK‐cell‐derived exosomes express CD56, NKG2D, KIR2DL2, FasL and perforin to kill cancer cells and educate other NK cells to perform cytotoxicity activity	^59^
Human NK cells	Ultracentrifuge	CD63(+) and TSG101(+)	Human pancreatic cell lines (MIA PaCa‐2 and PANC‐1)	Tumour inhibition	NK‐cell‐derived exosomal miR‐3607‐3p suppresses proliferation, migration and invasion by targeting IL‐26	^60^
Human NK cells	Size exclusion using 100 kDa MW cut‐off filter membrane	CD81(+), ALIX(+), TSG101(+), HSP70 (+) and Calnexin(–)	NB cell lines (CHLA‐136 and LAN‐5)	Tumour inhibition	NK‐cell‐derived exosomes deliver miR‐186 to inhibit MYCN‐amplified NB and prevent the TGFβ1‐dependent inhibition of NK cells by targeting CD56	^61^
NK‐92 MI cells	Density gradient ultracentrifugation	CD63(+), Alix(+) and GM130(–)	Murine melanoma cell line (B16F10l)	Tumour inhibition	NK‐92MI‐derived exosomes express FasL and perforin to kill melanoma cells	^62^
NK‐92 cells	Ultracentrifugation	CD63(+), CD81(+), cytochrome C(–) and α‐tubulin(–)	Human cancer cell lines (K562, Jurkat cells, A549 and HeLa)	Tumour inhibition	With IL‐15 and IL‐21 stimulation, NK‐92‐derived exosomes show enhanced cytotoxic capacity against tumour cells through CD226 (DNAM‐1) upregulation	^63^
NK‐92 cells	Ultracentrifugation	CD63(+), ALIX(+) and TSG101(+)	Breast cancer cell line (MCF‐7)	Tumour inhibition	PTX‐loaded NK‐92‐derived exosomes inhibit breast cancer cell proliferation and migration and induce tumour cell apoptosis through Bax and caspase‐3 upregulation	^64^
NK cells from healthy donors and lung cancer patients	Magnetic purification	CD56(+) and FLOT1(+)	Circulating tumour cells from NSCLC patients	Tumour inhibition	Healthy NK‐cell‐derived exosomes show cytotoxic effects against circulating tumour cells	^65^

Abbreviations: ATP, adenosine triphosphate; CAR‐T, chimeric antigen receptor T cell; CTL, cytotoxic T lymphocyte; DCs, dendritic cells; EBV, Epstein–Barr virus; EGFR, epidermal growth factor receptor; HER, human epidermal growth factor receptor; IFN‐γ, interferon‐gamma; IL, interleukin; MHC, major histocompatibility complex; MMP, matrix metalloproteinase; MSCs, mesenchymal stem cells; NA, not applicable; NB, neuroblastoma; NK, natural killer; NKL, NK‐cell‐enriched lymphocytes; NSCLC, non‐small cell lung cancer; PEG, polyethylene glycol; PTX, paclitaxel; TGFβ1, transforming growth factor beta 1; Th1, T helper 1; Treg, regulatory T cell.

## 3 MYELOID CELL‐DERIVED EXOSOMES

Myeloid cells are innate immune cells that include monocytes, macrophages, DCs and granulocytes. Under inflammatory conditions, the infiltrated tumour‐associated macrophages, tumour‐associated neutrophils and myeloid‐derived suppressor cells (MDSCs) exhibit immunosuppressive activity, creating a premetastatic niche for tumour cell implantation and are closely related to clinical outcomes in cancers.^66^

### 3.1 Tumour‐associated macrophage‐derived exosomes

Macrophage phenotype and function are dynamic and can change based on the microenvironment. Infection or tissue injury induces macrophage polarisation into the proinflammatory M1 phenotype, which produces proinflammatory factors such as IL‐6 and IL‐12 in combating pathogens. Subsequently, these cells polarise into the anti‐inflammatory M2 phenotype to repair damaged tissue.^67^ In the TME, M1 macrophages are generally considered antitumoural macrophages. In contrast, M2 macrophages mainly promote tumour outgrowth.^68^

With the antitumour potential of M1 macrophages, M1 macrophage‐derived exosomes (M1‐Exos) can activate immune responses, thereby combating tumour growth. Li et al. purified LPS‐ and IFN‐γ‐induced PBMCs, which exhibited M1 phenotypes, and showed that exosomal miR‐16‐5p suppressed PD‐L1 expression to enhance T‐cell immune responses to gastric cancer cells.^69^ Wang et al. showed that ferroptosis inducer RSL3‐loaded M1‐Exos prepared from murine bone marrow‐derived macrophages (BMDMs) promoted tumour cell death by ferroptosis, conferred an immunoreactive TME by reprogramming M2 macrophages to M1 phenotypes and increasing CD8(+) T‐cell infiltration.^70^ Wang et al. prepared the macrophage‐tumour hybrid cells by introducing tumour cells into macrophages for collecting chimeric exosomes. These chimeric exosomes showed elevated tumour accumulation, increased adaptive immunity and enhanced therapeutic efficacy combined with immune checkpoint blockade therapy (ICBT).^71^ The M1‐Exos from murine RAW264.7 cells displayed robust cytotoxicity against tumour cells by enhancing proinflammatory cytokine release from macrophages and caspase‐3 activities in cancer cells.^72^ Additionally, M1‐Exos can carry many tumour‐suppressive RNAs. Exosomal miR‐181a‐5p secreted by M1 macrophages inhibited ETS1, which repressed STK16 and subsequently abolished lung cancer cell growth.^73^ M1‐Exos carrying the long non‐coding transcript, HOXA transcript at the distal tip (HOTTIP), induced the TLR5/nuclear factor‐kappa B (NF‐κB) signalling pathway to suppress head and neck cancers by sponging miR‐19a‐3p and miR‐19b‐3p.^74^

For therapeutic purposes, Cheng et al. showed that M1‐Exos from M1‐polarised RAW246.7 cells were used as a potent immunopotentiator than CpG oligonucleotides to enhance the activity of lipid calcium phosphate nanoparticle‐encapsulated Trp2 vaccine.^75^ M1‐Exos also deliver drugs or natural extracts to enhance tumour eradication. Li et al. showed enhanced therapeutic effects using the macrophage‐derived exosome‐based c‐Met targeted drug delivery system, MEP‐D, against TNBC. The c‐Met binding peptides decorated the surface of macrophage‐derived exosomes, and the chemotherapeutic drug doxorubicin (DOX) was loaded in poly‐(lactic‐co‐glycolic acid) nanoparticles packed into the exosomes. These macrophage‐derived exosome‐coated particles showed improved tumour cytotoxicity and increased stability in the circulation.^76^ Docetaxel‐loaded M1‐Exos from RAW264.7 cells induced M1‐type polarisation of naïve macrophages and robust M1 activation in immunosuppressive TME.^77^ Zhou et al. showed exosome‐assisted bladder cancer treatment by loading a CD73 inhibitor (AB680) in macrophage‐derived exosome‐mimetic nanovesicles and adding an antibody targeting PD‐L1 on the exosome surface.^78^ Additionally, triptolide, a traditional Chinese herbal medicine with tumour‐killing activity, was loaded into macrophage‐derived exosomes with TRAIL engineered on the surface to promote apoptosis in melanoma cells.^79^

Pancreatic ductal adenocarcinoma is a highly lethal malignancy with limited efficacy in chemotherapy and immunotherapy and exhibits poor survival rates attributed to the malignant TME featured with protumoural macrophages and fibroblasts.^80^ Kamerkar et al. generated normal foreskin fibroblast‐derived exosomes carrying CD47 to prevent phagocytosis as carriers to deliver siRNA targeting KrasG12D to inhibit tumour outgrowth.^81^ The anticancer effects of exosomal miR‐124 of bone marrow (BM)‐MSCs,^82^ exosomal miR‐145‐5p from human umbilical cord mesenchymal stromal cells^83^ and exosomal miR‐3607‐3p from NK cells^60^ have been reported. Moreover, Yin et al. showed that exosomal miR‐501‐3p was secreted by M2 macrophages and targeted TGFBR3 in pancreatic cancer, resulting in cell proliferation, angiogenesis and metastasis and inhibiting cell apoptosis. Inhibition of exosomal miR‐501‐3p by transfecting antagomiR in macrophage exosomes repressed tumour formation.^84^ In esophageal cancer, M2 macrophage‐derived exosomes (M2‐Exos) contain the lncRNA AFAP1‐AS1, which activates ATF2 by sponging miR‐26a to mediate invasion and metastasis.^85^ In medulloblastoma, M2‐Exos encapsulating miR‐155‐3p increased the invasiveness of medulloblastoma by inhibiting the expression of WD repeat domain 82.^86^ M2 macrophages transferred exosomal miR‐155‐5p and miR‐221‐5p to endothelial cells to enhance angiogenic activities by inhibiting E2F2, promoting the growth of pancreatic tumours.^87^ Exosomal miR‐21‐5p and miR‐155‐5p in M2‐Exos also promoted the mobility, migration and invasion of colon cancer cells by downregulating BRG1.^88^

M2‐Exos orchestrate an immunosuppressive TME. M2‐Exos inhibited the expression of ZC3H12B in colon cancer cells through miR‐155‐5p, in turn, increased the expression of IL‐6, resulting in a decrease in CD3(+) T cells and IFN‐γ(+) T cells.^89^ In liver cancer, exosomal miR‐21‐5p derived from M2 macrophages suppressed YOD1 expression and increased the expression of Yes‐associated protein and β‐catenin, resulting in CD8(+) T‐cell exhaustion.^90^ Zheng et al. showed that exosomal ApoE from M2‐Exos suppressed the ATPase activity of BiP, resulting in decreased MHC‐I expression, thereby resisting ICBT therapy.^91^ Cianciaruso et al. showed that tumour‐associated macrophage‐derived exosomes (TAM‐Exos) and cancer cell‐derived exosomes were the main cellular sources of exosomes in a syngeneic MC38 colon cancer model. TAM‐Exos isolated with an anti‐CSF1R exhibited an M1‐like tumour‐associated macrophage (TAM) signature and promoted T‐cell proliferation even though the TAM cells themselves exhibited an M2‐like phenotype.^92^ M2‐Exos could also be used for therapy in non‐cancer diseases. M2‐Exos collected from M2‐polarised BMDMs were taken up by M1 macrophages and promoted the M1‐to‐M2 macrophage polarisation, thereby accelerating diabetic fracture healing in vivo.^93^

Although the adoptive cell transfer by CAR‐T and CAR‐NK has made significant progress, insufficient infiltration into solid tumour sites is a major hurdle. With tumour site abundance and infiltration, CAR‐M are focused on immunotherapy.^8^ CAR‐M primarily relies on CAR's antigen‐targeting ability to enhance the binding of macrophages to cancer cells expressing CD19 and to eliminate cancer cells through phagocytosis.^94^ Huo et al. showed that the murine M1 CAR‐M targeting human epidermal growth factor receptor (HER) exhibited enhanced antigen presentation capabilities and the secretion of proinflammatory cytokines.^95^ Klichinsky et al. also demonstrated that human M1 CAR‐M targeting HER performed antigen‐specific phagocytosis and maintained the M1 phenotype for sustaining proinflammatory TME and antitumour T‐cell activation.^96^ To optimise the CAR‐M efficacy, Zhang et al. developed the induced pluripotent stem cell‐derived CAR‐M (CAR‐iMac) exhibiting greater cell survival and expansion ability as potentially universal cells for cancer therapy.^97^ Kang et al. reprogrammed macrophages to M1 CAR‐M in vivo to reduce the complexity of CAR‐M production ex vivo and cell‐manufacturing costs.^98^ With the advance in the cultivation and expansion of CAR‐M, we foresee active engagement of CAR‐M‐derived exosomes in cancer therapeutics.

### 3.2 Neutrophil‐derived exosomes

Apart from macrophages, Fridlender et al. classified the polarisation state of granulocytic neutrophils into the N1 and N2 phenotypes based on the nomenclature of M1/M2 macrophages.^99^ N1 neutrophils promote immune responses by increasing the production of molecules such as tumour necrosis factor (TNF), reactive oxygen species (ROS) and Fas and exhibited an antitumourigenic N1 phenotype. Conversely, N2 neutrophils have increased expression of protumoural factors, promote tumour growth and are protumourigenic N2 phenotypes associated with poor tumour prognosis.^100^ Thus, N2‐neutrophil‐derived exosomes (N2‐Exos) demonstrate protumoural activity. A study indicated that nicotine‐induced neutrophil N2 polarisation led to the secretion of miR‐4466‐containing N2‐Exos to promote lung cancer growth and brain metastasis.^101^

tExos can be made from neutrophils. Wang et al. prepared the DOX‐loaded neutrophil exosomes and found that these exosomes penetrated the blood–brain barrier and responded chemotactically to inflamed brain tumours to improve glioma therapy.^102^ Exosome‐mimetic nanovesicles (NVs) are advantageous in drug delivery as the production efficiency of NVs is 100 times greater than that of exosome purification in the culture system and show biocompatibility equivalent to that of exosomes with less immunogenicity.^103^ Zhang et al. loaded DOX into exosome‐like nanovesicles derived from neutrophils (NNVs) and decorated them with magnetic superparamagnetic iron oxide nanoparticles (SPIONs). In a magnetic field, the SPION‐NNV‐DOX platform effectively targeted regional tumours and induced tumour apoptosis without harming healthy organs, providing a safe and effective cancer treatment option.^104^

### 3.3 Myeloid‐derived suppressor cell‐derived exosomes

MDSCs are heterogeneous cells composed of myeloid progenitor cells and immature myeloid cells. There are two groups of MDSCs in humans and mice: polymorphonuclear MDSCs and monocytic MDSCs. Although MDSCs show phenotypic markers of neutrophils and monocytes, their activation process, maturity and developmental factors differ.^105^

**FIGURE 2:**
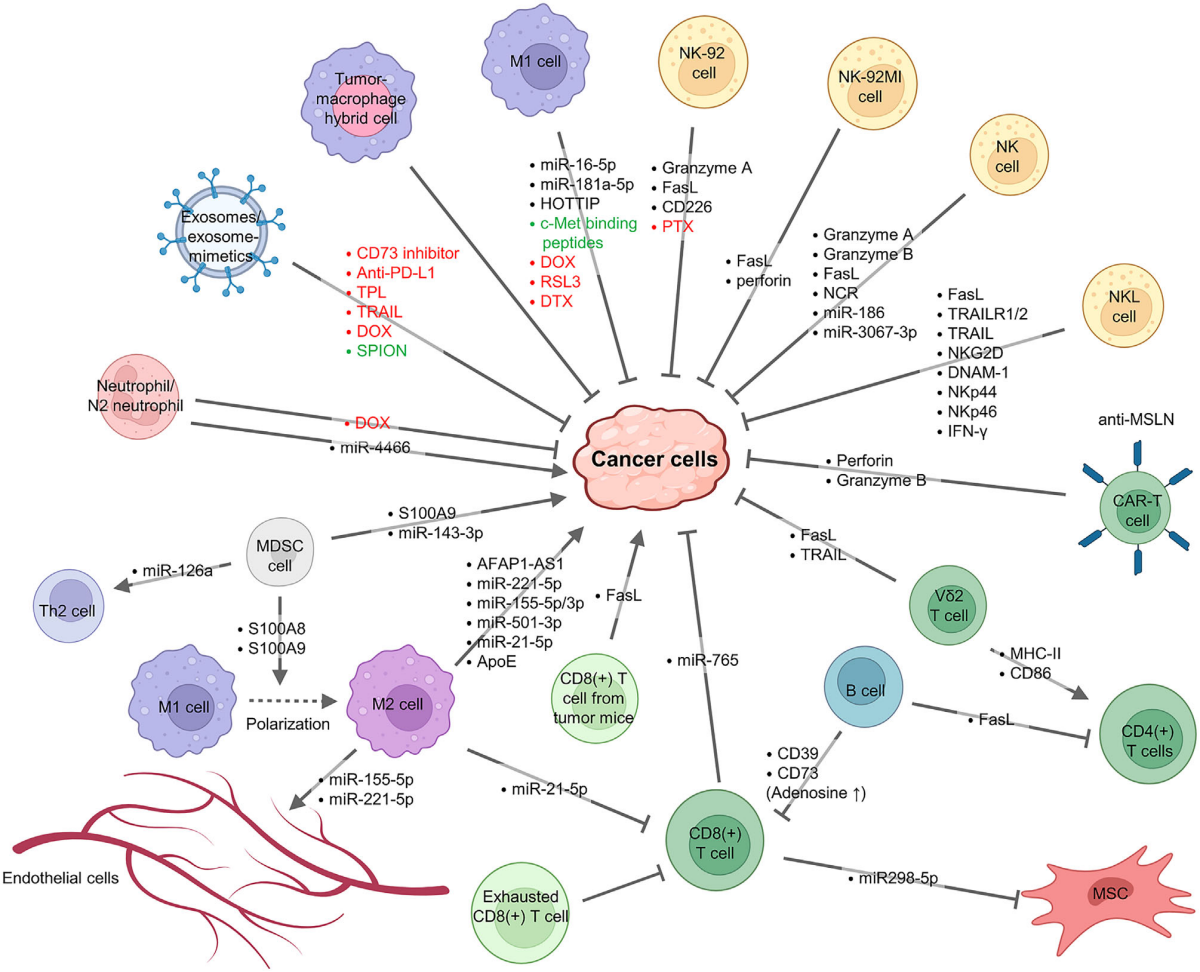
Impacts of immune cell‐derived exosomes and exosome mimetics on cancer progression. Exosomes secreted by immune cells can promote or inhibit cancer cell progression. B cells, M2 macrophages, N2 neutrophils and myeloid‐derived suppressor cells (MDSCs) mainly produce exosomes that promote cancer cell development. Exosomes released from natural killer (NK) cells, CD4(+) T cells, CD8(+) T cells, M1 macrophages and exosome mimetics derived from neutrophils suppress cancer development. Exosomal components, exosome‐encapsulated therapeutic agents and engineered targeting molecules on exosomes are labelled in black, red and green, respectively. The figure is created using BioRender. DOX, doxorubicin; MSCs, mesenchymal stem cells; MSLN, mesothelin; N2, N2 neutrophils; NKL, natural killer cell‐enriched lymphocytes; PTX, paclitaxel; Th2, T helper 2; TPL, triptolide.

Under hypoxic conditions, granulocytic MDSC (G‐MDSC)‐derived exosomes carrying S100A9 proteins can promote cancer stemness in murine CT26 colon cancer cells and azoxymethane/dextran sodium sulfate (AOM/DSS)‐induced colon cancer.^106^ Exosomal S100A8 and S100A9 on Gr1(+) MDSCs from a 4T1 syngeneic breast tumour model promoted M2 macrophage polarisation.^107^ Administration of 4T1‐bearing mice with the chemotherapy drug DOX promoted the release of IL‐33 from breast cancer cells and the induction of IL‐13(+) Th2 cells and IL‐13R(+)miR‐126a(+) MDSCs in a feedforward manner. Exosomes from DOX‐treated Gr1(+) MDSCs promoted angiogenesis and inhibited T‐cell activity, thereby facilitating lung metastasis.^108^ Gr1(+) MDSC‐derived exosomes modulated the TME by inducing the expression of protumourigenic factors, leading to a decrease in proinflammatory M1 macrophages, CD8 T‐cell exhaustion, and an increase in ROS production that led to CD8 T‐cell apoptosis. This process ultimately inhibited the immune response and promoted tumour growth and invasion.^109^ Exosomal miR‐143‐3p from G‐MDSCs downregulated integral membrane protein 2B and enhanced the PI3K/Akt signalling for lung cancer progression.^110^ Therefore, suppressing MDSC exosome‐mediated TME modulation may be essential for cancer therapy. The impacts of myeloid cell‐derived exosomes on TME modulation are summarised in Table 2. Exosomal‐driven intercellular communications among immune and cancer cells are shown in Figure 2.

**TABLE 2:** Functions of myeloid cell‐derived exosomes in the tumour microenvironment (TME).

Cell source	Exosome isolation	Verified exosome markers	Recipient cells	Disease status	Molecules of actions	Reference
Human M1‐polarised macrophages	Ultracentrifugation	CD63(+)	Human gastric cancer cell lines (AGS and NCI‐N87)	Tumour inhibition	M1‐macrophages‐derived exosomal miR‐16‐5p downregulates PD‐L1 on cancer cells	^69^
Murine M1 macrophages from BMDMs	Ultracentrifugation	CD9(+), CD63(+), CD81(+), ALIX(+) and Calnexin(–)	Murine breast cancer cell line (4T1) and murine colon cancer cell line (CT26)	Tumour inhibition	RSL3‐loaded M1‐Exos induce tumour cell death by ferroptosis, accompanied by alleviating immunosuppressive TME by promoting M2 macrophages polarised into M1 type, decreasing Tregs and increasing CD8(+)T cells numbers	^70^
Macrophage‐tumour hybrid cells	Gradient centrifugation and ultracentrifugation	CD9(+), TSG101(+) and Alix(+)	Murine breast cancer cell line (4T1), melanoma cell line (B16), and human breast cancer cell line (MDA‐MB‐231)	Tumour inhibition	Chimeric exosomes are internalised by APCs function and serve as antigen‐presenting particles to stimulate T‐cell activity. Chimeric exosomes confer M1‐type polarisation and reduce Tregs for establishing antitumoural TME	^71^
Murine macrophage RAW264.7 cells	Ultracentrifugation	CD9(+), TSG101(+) and Alix(+)	Murine breast cancer cell line (4T1)	Tumour inhibition	M1‐Exos stimulate naïve macrophages to increase proinflammatory cytokines release through the NF‐κB pathway and increase the activities of caspase‐3 in cancer cells. Paclitaxel‐loaded M1‐Exos displayed robust cytotoxicity against tumour cells	^72^
Human THP‐1 monocytic cells	Ultracentrifugation	ALIX(+), CD63(+) and TSG101(+)	Human lung cancer cell lines (H1299 and H1975 cell)	Tumour inhibition	M1‐macrophages‐derived exosomal miR‐181a‐5p inhibits STK16 expression by targeting ETS1 to promote apoptosis in cancer cells	^73^
Human THP‐1 monocytic cells	Exosome precipitation kit	CD9(+) and CD63(+)	Human head and neck squamous cell carcinoma cell lines (FaDu, CNE‐2Z and Hep‐2)	Tumour inhibition	M1‐macrophage‐derived exosomal lncRNA HOTTIP suppresses cancer cell growth through upregulation of the TLR5/NF‐κB signalling pathway by sponging miR‐19a‐3p and miR‐19b‐3p	^74^
Murine macrophages RAW264.7 cells	Size exclusion using 200 kDa MW cut‐off filter membrane followed by ultracentrifugation	NA	Mouse macrophages and DCs	Tumour inhibition	M1 macrophage‐derived exosomes induce the proinflammatory cytokine secretion to stimulate Th1 cell activation	^75^
Murine macrophages RAW264.7 cells	Ultracentrifugation	CD63(+) and CD81(+)	Human breast cancer cell line (MDA‐MB‐231)	Tumour inhibition	The c‐Met targeting macrophage‐derived exosomes loaded with DOX‐containing polymeric nanoparticles is used for cancer therapy	^76^
Murine macrophages RAW264.7 cells	Ultracentrifugation	NA	Murine breast cancer cell line (4T1)	Tumour inhibition	DTX‐loaded in M1‐Exos promote M1 polarisation and inhibit tumourigenesis	^77^
Murine macrophages RAW264.7 cells	Serial cell extrusion followed by ultracentrifugation	NA	Murine bladder cancer cell line (MB49)	Tumour inhibition	Macrophage‐derived exosome‐mimetic nanovesicles loaded with the CD73 inhibitor and the anti‐PD‐L1 antibody reduce extracellular adenosine production and promote the activation of cytotoxic T cells, respectively	^78^
Murine macrophages RAW264.7 cells	Ultracentrifugation	CD9(+), CD63(+) and TSG101(+)	Human melanoma cell line (A375)	Tumour inhibition	Macrophage‐derived TRAIL‐engineered exosomes loaded triptolide for cancer targeted therapy	^79^
Human THP‐1 monocytic cells	Ultracentrifugation followed by exosome precipitation kit	TSG101(+), CD63(+) and CD81(+)	Human pancreatic cell lines (PANC‐1, BxPC‐3, MIA PaCa‐2 and Capan‐2)	Tumour progression	M2 macrophage‐derived exosomal miR‐501‐3p inhibits tumour suppressor TGFBR3 gene and facilitates cancer development by activating the TGF‐β signalling pathway	^84^
Human M2 macrophages	Ultracentrifugation	CD63(+), CD81(+), TSG101(+) and GRP94(+)	Human esophagus cancer cell line (KYSE410)	Tumour progression	M2 macrophage‐derived exosomal AFAP1‐AS1 downregulates miR‐26a and promotes the expression of ATF2 in cancer cells	^85^
Murine M2 macrophages	Ultracentrifugation	CD81(+), ALIX(+) and TSG101(+)	Human medulloblastoma cell lines (Daoy, D283, ONS‐76 and D341)	Tumour progression	miR‐155‐3p‐loaded M2 macrophage‐derived exosomes enhance the growth of medulloblastoma cells by downregulating WDR82	^86^
Murine M2 macrophages	Ultracentrifugation followed by exosome precipitation kit	CD9(+) and CD63(+)	Murine aortic endothelial cells	Tumour progression	M2 macrophage‐derived exosomes carry miR‐155‐5p and miR‐221‐5p to endothelial cells, promoting angiogenesis by targeting E2F2	^87^
Human M2 macrophages from cancer tissues	Ultracentrifugation	CD63(+) and CD81(+)	Human colon cancer cell lines (SW48 and SW480)	Tumour progression	M2‐Exos transfer miR‐21‐5p and miR‐155‐5p into cancer cells to promote mobility, migration and invasion by downregulating BRG1	^88^
Human M2 macrophages	Ultracentrifugation	CD63(+) and CD81(+)	Human colon cancer cell (SW48)	Tumour progression	M2 macrophage‐derived exosomal miR‐155‐5p reduced the ZC3H12B expression to upregulate IL‐6 for immune escape	^89^
Murine M2 macrophages	Ultracentrifugation	CD9(+), CD63(+) and CD81(+)	Murine CD8(+) T cells	Tumour progression	M2 macrophage‐derived exosomal miR‐21‐5p inhibits YOD1 expression and activates the YAP/β‐catenin pathway in CD8(+) T cells for T‐cell exhaustion	^90^
M2 macrophages from BMDMs and human THP‐1 cells	Ultracentrifugation	CD63(+), CD81(+) and TSG101	Murine colon adenocarcinoma cell lines (MC38), and human gastric cancer cell lines (BGC‐823 and MGC‐803)	Tumour progression	Enrichment of ApoE in cancer cells transferred from M2 macrophage‐derived e‐Exos targets BiP in the endoplasmic reticulum, resulting in decreased MHC‐I expression	^91^
Nicotine‐polarised mouse N2‐neutrophils	Ultracentrifugation	NA	Lung cancer‐bearing mouse (H2030BrM and LL/2)	Tumour progression	Exosomal miR‐4466 secreted from nicotine‐induced N2‐neutrophil increases stemness and activates the SOX2/CPT1A pathway to promote cancer metastasis	^101^
Murine neutrophils	Ultracentrifugation	ALIX(+), CD63(+), TSG101(+) and Flotillin‐1(+)	Zebrafish models and murine glioma cell line (C6)	Tumour inhibition	DOX‐loaded neutrophils can enter the brain and target glioma	^102^
Human U937 monocytic cells and murine macrophages RAW264.7 cells	Serial cell extrusion followed by density ultracentrifugation	CD63(+) and TSG101(+)	HUVECs	Tumour inhibition	Chemotherapeutic drug‐loaded nanovesicles induce TNF‐α‐stimulated endothelial cell death in a dose‐dependent manner	^103^
Human neutrophils	Ultracentrifugation	ALIX(+), CD9(+), CD63(+) and CD81(+)	Human gastric/colon/hepatocellular cancer cell lines (HGC27, SW480 and HepG2)	Tumour inhibition	Human neutrophil‐secreted exosomes are modified with SPIONs and loaded with DOX to target and eliminate tumour cells	^104^
Murine and human G‐MDSCs	Exosome precipitation kit	CD9(+) and CD63(+)	Colon cancer cell lines (CT‐26 and SW480)	Tumour progression	G‐MDSC‐derived exosomal S100A9 induces the stemness of colon cancer cells	^106^
Murine MDSCs	Ultracentrifugation	Annexins, CD177, VSP35, Hsp70 and Hsp90	Murine breast cancer cell line (4T1)	Tumour progression	The proinflammatory proteins S100A8 and S100A9 are abundant in MDSC‐derived exosomes, which polarise macrophages towards a tumour‐promoting phenotype	^107^
Murine MDSCs	Ultracentrifugation followed by exosome precipitation kit	NA	Murine Th2 cells	Tumour progression	DOX treatment promotes the induction of IL‐13(+) Th2 cells for promoting the production of IL‐13R(+)/miR‐126a(+) MDSCs and miR‐126a(+) MDSC‐exosomes. Exosomal miR‐126 prevents DOX‐induced MDSC death in S100A8/A9 manner	^108^
Murine MDSCs	Ultracentrifugation	NA	CD8(+) T cells	Tumour progression	MDSC‐derived exosomes contain Fas and TNF‐1α for increasing ROS production and inciting the Fas/FasL pathway in CD8(+) T cells, leading them to apoptosis	^109^
Murine G‐MDSCs	Ultracentrifugation	CD63(+) and TSG101(+)	Human lung cancer cell lines (LTEP‐a‐2, SPC‐A‐1, and A549) and mouse lung cancer cell line (LLC)	Tumour progression	Exosomal miR‐143‐3p from G‐MDSCs promotes the proliferation of lung cancer cells by targeting ITM2B	^110^

Abbreviations: APC, antigen‐presenting cell; BMDMs, bone marrow‐derived macrophages; DCs, dendritic cells; DOX, doxorubicin; DTX, docetaxel; G‐MDSC, granulocyte MDSC; HOTTIP, HOXA transcript at the distal tip; HUVECs, human umbilical vein endothelial cells; IL, interleukin; ITM2B, integral membrane protein 2B; MDSCs, myeloid‐derived suppressor cells; MHC, major histocompatibility complex; M1‐Exos, M1 macrophage‐derived exosomes; M2‐Exos, M2 macrophage‐derived exosomes; NA, not applicable; PD‐L1, programmed death‐ligand 1; ROS, reactive oxygen species; SPIONs, superparamagnetic iron oxide nanoparticles; Th1, T helper 1; TNF, tumour necrosis factor; TRAIL, tumour necrosis factor‐related apoptosis‐inducing ligand; Tregs, regulatory T cells; WDR82, WD repeat domain 82; YAP, Yes‐associated protein.

### 3.4 Platelet‐derived microvesicles and exosomes

Platelets derived from megakaryocytes are anucleate cells that play crucial roles in blood clotting and vascular repair.^111^ Adhesion molecules such as P‐selectin, glycoprotein Ib and junctional adhesion molecule A allow platelets for host–cell interactions during inflammation.^112^ The roles of platelets in cancer progression have been highlighted. CTCs interact with activated platelets and leukocytes, thereby generating aggregates to facilitate endothelial attachment. The activated platelets and fibrins covering cancer cells protect cancer cells from NK‐cell‐mediated cytotoxicity.^113^ TREM‐like transcript 1 is secreted from platelets in non‐small cell lung cancer (NSCLC) patients and further binds to CD3ε on CD8 (+) T cells to inhibit T‐cell activity, subsequently promoting immune evasion by cancer cells.^114^ With the involvement of platelets in cancer progression, thrombocytosis (>350 × 10^9^/L) has been shown to be correlated with the unfavourable progression of ovarian cancer.^115^ Moreover, platelet RNA profiles are used to detect cancer^114^ and predict cancer progression.^116^

Platelet‐derived EVs contribute to the formation of blood clots by displaying EV surface receptors that interact with coagulation factors. These receptors comprise GPIIb/IIIa, which mediates platelet aggregation; tissue factor (TF), which initiates the extrinsic pathway of coagulation; and phosphatidylserine, which facilitates the activation of factors II and X, contributing to the generation of thrombin and fibrin.^117^, ^118^ Treatment of human pulmonary microvascular endothelial cells with platelet‐derived exosomes ameliorated the thrombin‐mediated loss of VE‐cadherin and ZO‐1 expression in endothelial cells.^119^ The platelet exosomal miR‐126 promotes capillary tube formation.^120^ However, thrombin‐activated platelets released miR‐223‐ encapsulated exosomes to inhibit the expression of ICAM‐1 by inhibiting the NF‐κB and MAPK signallings in recipient human umbilical vein endothelial cells treated with TNF‐α.^121^ Exosomes from platelets stimulated with the nitric oxide (NO) donor diethylamine‐NONOate or LPS mimicked diseased exosomes from septic patients and carried type II NO synthase and NADPH oxidase, thereby promoting ROS production and caspase‐3‐mediated apoptosis in the rabbit aorta endothelial cells.^122^ Platelet‐derived microparticles (PMPs), which are mainly MVs, also regulate the activity and adhesion of stem cells and progenitor cells. Haematopoietic stem and progenitor cells treated with thrombin‐activated platelet‐derived exosomes showed no significant changes in clonogenicity but increased adhesion to endothelial cells. Murine BM cells decorated with PMPs engrafted in irradiated mice faster than those not decorated, suggesting the critical roles of PMPs in optimising transplantations.^123^ Platelets can transfer specific receptors (CD41, CD62 and CXCR4) to the membranes of immature blast cells through PMPs. This process activates MAPK, PI3K–AKT and STAT signalling, thereby enhancing chemotaxis and adhesion to fibrinogen and endothelial cells and promoting cell survival.^124^

Platelet‐secreted MVs (PMVs) and exosomes also play critical roles in cancer progression. Janowska‐Wieczorek et al. isolated PMVs and exosomes from thrombin‐ and collagen‐activated platelets and found that PMVs promoted the proliferation of lung cancer cells (A547, HTB177, HTB183 and CRL2062) and promoted the expression of angiogenic genes, including vascular endothelial growth factor, IL‐8 and hepatocyte growth factor. Treatment with PMVs increased the expression of MMP‐2 and MMP‐9 in A549 cells and MT1‐MMP in HTB177 cells and increased the phosphorylation of MAPK and AKT (A547, HTB177, CRL2062 and CRL2066). Platelet‐derived exosomes also promoted the growth of A549 cells. PMV‐treated murine Lewis lung carcinoma cells also exhibited enhanced metastasis.^125^ PMVs transferred integrin GPIIb/IIIa (CD41), which is known to drive tumour metastasis,^126^ to the surface of breast cancer cells (MDA‐MB‐231 or BT‐549), increased their adhesion to endothelial cells, increased chemotaxis, and stimulated the production of MMPs to promote cancer progression.^127^ Exosomes from K562‐derived megakaryocytic cells and murine platelets carry HMGB1, inhibiting DOX‐induced caspase‐3 activity and reducing apoptosis in human bladder carcinoma T24 cells and murine lewis lung carcinoma (LLC) cells, ultimately promoting cancer cell survival.^128^

Platelets can be educated by cancer cells. Dudiki et al. suggested that cancer exosomes secreted from LNCaP‐C4‐2 prostate cancer cell lines were enriched with N‐glycosylated CD63 on the surface, which interacted with RPTPα on the platelet surface and increased the phosphorylation of Src, Akt and Erk, thereby activating platelets and accelerating thrombosis formation.^129^ Moreover, Wysoczynski et al. showed that the surface expression of TF on rhabdomyosarcoma (RMS) cells promoted thrombin activation, subsequently stimulating the secretion of PMVs. Thrombin‐activated PMVs could transfer platelet GPIIb/IIIa (CD41) to RMS cells. PMVs also promote the phosphorylation of MAPK and AKT, enhancing chemotaxis and adhesion in RMS cells. Additionally, thrombin can directly interact with PAR‐1 and PAR‐3 on RMS cells, decreasing local chemoattractants and adhesion of RMS cells and facilitating the release of RMS cells into circulation.^130^ Moreover, cancer exosomes from MDA‐MB‐231 breast cancer cells and thrombin‐activated platelets reduced invasion and increased apoptosis in Jurkat T cells, thereby leading to immune escape in a dynamic TME.^131^

**FIGURE 3:**
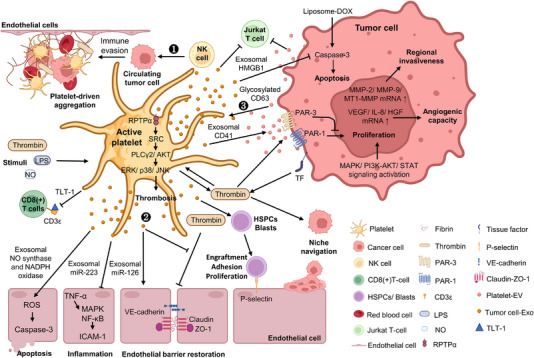
Roles of platelets and platelet‐derived extracellular vesicles in cancer progression. (1) Platelets interact with tumour cells and endothelial cells to form cell aggregates, thereby facilitating metastasis and immune evasion. (2) Platelet‐derived microvesicles and exosomes stimulate the proliferation and angiogenic capacities of educated cancer cells. (3) Platelets are also educated by cancer exosomes to facilitate coagulation, favouring a metastatic tumour microenvironment (TME). The figure was created using BioRender. DOX, doxorubicin; EV, extracellular vesicle; HSPC, haematopoietic stem/progenitor cells; LPS, lipopolysaccharide; NK, natural killer; NO, nitric oxide; ROS, reactive oxygen species; TF, tissue factor.

Drug‐loaded platelet‐derived exosomes can be prepared for cancer therapy by drug incubation, sonication, electroporation, extrusion or dialysis.^132^ Uslu et al. found that exosomes from adenosine diphosphate‐activated platelets promoted breast cancer cell growth (MDA‐MB‐231). When DOX was delivered into platelet‐derived exosomes through electroporation, reduced recipient cancer cell viability was observed.^133^ Ying et al. showed that DOX‐ or vancomycin‐loaded exosome‐like platelet nanovesicles could be delivered to tumour sites, exhibiting superior tumour treatment efficacy and prolonging survival.^134^ The platelet‐based artificial nanovesicle TRAIL‐DOX‐PM‐NVs contain platelet membranes modified with TRAIL protein on the surface and a DOX‐loaded nanogel‐based inner core. TRAIL‐DOX‐PM‐NV effectively accumulated at the tumour site by binding P‐selectin on the nanovesicle to mucin cancer cell surfaces to enhance TRAIL‐induced apoptosis.^135^ MV and exosome‐based platelet–tumour cell interactions are shown in Figure 3.

### 3.5 RBC‐derived exosomes as therapeutic vehicles

RBC‐derived exosomes (RBC‐Exos) are crucial in immune regulation and human disease. RBC‐Exos stimulated the activity of PBMCs to induce the generation of inflammatory cytokines and prolonged the lifetime of unactivated PBMCs, thereby enhancing CD4(+) and CD8(+) T‐cell activity. Elucidating the functions of RBC‐Exos in blood transfusion products could benefit transfusion recipients by augmenting their immunity.^136^ The sensitisation of PB monocytes in Parkinson's disease (PD) patients is closely associated with disease severity.^137^ RBC‐Exos isolated from PD model mice contained α‐synuclein and stimulated monocyte hyperactivation through LRRK2 activation.^138^ In malaria, infected RBCs release higher amounts of exosome‐like vesicles than uninfected cells. These exosome‐like vesicles facilitated the differentiation of *Plasmodium falciparum* into sexual forms by transmitting plasmids at the ring stage, thereby promoting parasite survival and growth.^139^

Due to the abundance of RBCs in circulation and their availability in the blood, RBC‐Exos can readily be prepared for downstream applications. The lack of nuclei and mitochondrial DNA in RBCs makes RBC‐Exos an ideal exogenous carrier for nucleic acid drugs. Usman et al. used group O RBC‐Exos as universal agents for horizontal delivery of RNA drugs such as antisense oligonucleotides (ASOs), Cas9 mRNA and guide RNAs. They demonstrated that RBC‐Exos robustly suppressed miRNAs and Cas9‐mediated genome editing against a broad spectrum of cancers without discernable side effects.^140^ In a recent study, RBC‐Exos were used as hydrophobic carriers of iron oxide particles for delivery into human BM‐MSCs, enabling the tracking of MSCs by magnetic resonance imaging without compromising gene expression and cell viability.^141^ Due to the predisposition of hepatic accumulation of RBC‐Exos,^140^ Zhang et al. electroporated miR‐155‐targeting ASOs and chemical drugs, including DOX and sorafenib into RBC‐Exos to treat liver cancer.^142^

**FIGURE 4:**
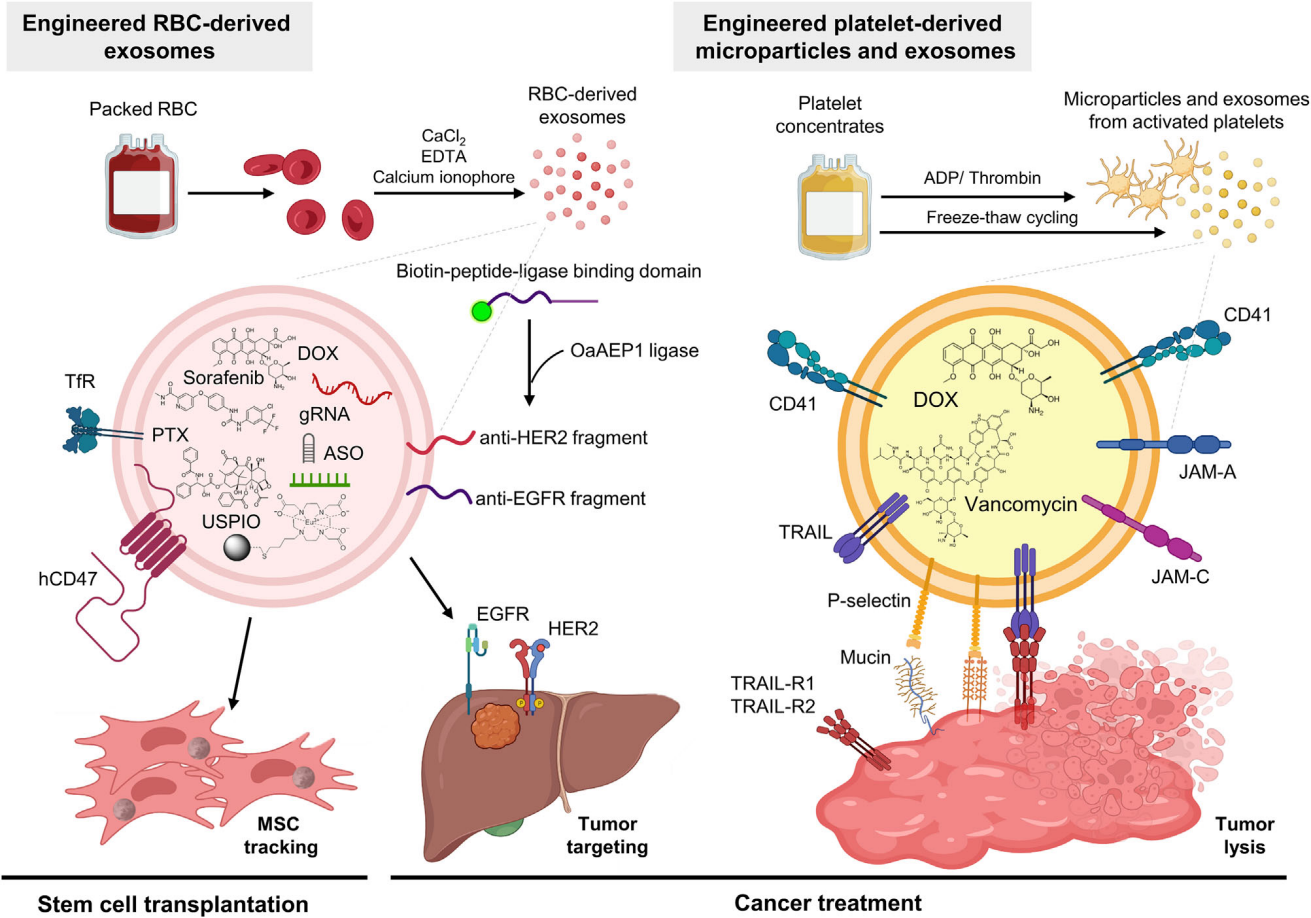
Engineering exosomes and exosome mimetics derived from platelets and red blood cells (RBCs) for targeted cancer therapy. Platelets and RBCs are standard blood products prepared for transfusion. Exosomes can be collected and used to prepare packed RBCs and platelet concentrates for chemical or physical engineering to improve cancer cell targeting and can be loaded with anticancer agents to enhance therapeutic efficacy. In chemical modifications, the anti‐human epidermal growth factor receptor 2 (HER2) and anti‐epidermal growth factor receptor (EGFR) fragments were ligated through an OaAEP1 ligase, and tumour necrosis factor‐related apoptosis‐inducing ligand (TRAIL) was attached through the sulphosuccinimidyl‐4‐(N‐maleimidomethyl)cyclohexane‐1‐carboxylate (sulpho‐SMCC) linkage. The anticancer drugs were loaded into exosomes by electroporation or sonication in physical modifications. The tumour‐inhibiting oligonucleotides were transfected or electroporated into exosomes. The delivery of ultrasmall superparamagnetic iron oxide nanoparticles (USPIO) into exosomes was achieved by hypo‐osmotic solution mixing. The figure was created using BioRender. ADP, adenosine diphosphate; ASO, antisense oligonucleotide; CI, calcium ionophore; DOX, doxorubicin; EDTA, ethylenediaminetetraacetic acid; EGFR, epidermal growth factor receptor; gRNA, guide RNA; JAM, junctional adhesion molecule; MSC, mesenchymal stem cell; PTX, paclitaxel; TfR, transferrin receptor; TRAIL‐R, TRAIL receptor.

The antitumoural potential of RBC‐Exos with tumour specificity has been uncovered. As it is challenging to express foreign proteins in RBCs due to the absence of ribosomes, an alternative approach is modifying and engineering RBC‐Exos. Pham et al. showed an enzymatic methodology for the covalent binding of peptides or nanobodies to exosomes. Specifically, group O RBC‐Exos from healthy donors were isolated and stimulated to induce exosome release in the presence of a calcium ionophore. RBC‐Exos were conjugated with anti‐epidermal growth factor receptor and anti‐HER2 nanobodies via the OaAEP1 ligase. The loading of PTX through sonication resulted in increased therapeutic efficacy and efficiency.^143^ Rodriguez et al. further showed that the incorporation of minimal human‐CD47‐based peptide as a self‐peptide signal in RBC‐Exo reduced the uptake of engineered RBC‐Exos by monocyte cell lines, resulting in a prolonged half‐life of RBC‐Exos.^144^ However, the roles of RBC‐Exos in cancer progression remain unclear. We summarised the use of engineered exosomes derived from platelets and RBCs in Figure 4.

## 4 CLINICAL TRIALS ON BLOOD CELL‐DERIVED EV IN HUMAN DISEASE

The clinical application of exosomes derived from blood cells is currently limited. As of 2023, four studies on the use of blood‐derived exosomes are documented in the ClinicalTrials.gov database (Table 3). In NCT02594345, the research team conducted mixed exosomes secreted from RBCs from healthy individuals in vitro. Thromboelastometry was used to assess the impact of RBC‐Exos on clotting to understand the roles of RBC‐Exos in blood coagulation. There are two clinical studies related to DC‐derived exosomes (DC‐Exos). One study compared the level of exosomes obtained from patients with sepsis and healthy individuals and identified the differentially expressed exosomal miRNAs as diagnostic biomarkers (NCT02957279). In the clinical trial NCT01159288, a cohort of 47 patients with advanced unresectable NSCLC was enrolled to investigate the effectiveness of immunotherapy combining metronomic cyclophosphamide therapy with DC‐Exos. The researchers found that DC‐Exos prolonged patient PFS by enhancing NKp30‐dependent NK‐cell function.^145^ One related study on melanoma indicated the ability of DC‐Exos to stimulate NK cells.^146^ Treating 13 patients with unresectable stage III or IV NSCLC with DC‐Exos did not result in significant organ toxicity, and no autoimmune reactions were observed, suggesting the efficacy and safety of DC‐Exos.^147^

**TABLE 3:** Current clinical trials on blood cell‐derived exosomes.

Number	Official title	Trail types	Interventions	Participants	Status	Completion date
NCT02594345	In vitro study of the effect of exosomes derived from red blood cell units on platelet function and blood coagulation in healthy volunteers' blood	Diagnosis	In vitro assay	18	Completed	December 2015
NCT04389385	Aerosol inhalation of the exosomes derived from allogenic COVID‐19 T cells in the treatment of early‐stage novel coronavirus pneumonia	Therapeutics	Therapeutic exosome inhalation	60	Unknown^a^ (phase I)	31 May 2021
NCT02957279	A cohort study to investigate the impacts of peripheral blood dendritic cells‐derived exosomes at an early phase on the prognosis in human sepsis	Prognostics	Prospective cohort study	50	Unknown	October 2017
NCT01159288	Phase II trial of vaccination with tumour antigen‐loaded dendritic cell‐derived exosomes on patients with unresectable non‐small cell lung cancer responding to induction chemotherapy	Therapeutics	Therapeutic exosome injection	41	Completed (phase 2)	19 December 2015

^a^ Unknown; the study has passed its completion date, and its status has not been verified in more than 2 years.

## 5 FUTURE PERSPECTIVES AND PRACTICAL CONSIDERATIONS

Exosome‐based therapy is a promising strategy to complement or augment CAR‐T/NK/M therapy. As discussed earlier, using exosomes derived from CAR‐T instead of CAR‐T itself may have the benefit of lowering the risk of CRS. The CAR‐T‐derived exosome therapy may also be simplified compared to the use of CAR‐T cells since maintaining the therapeutic agent's bioactivity is less of a concern with proper storage and delivery guidelines. However, ensuring the quality of CAR‐T/NK/M‐derived exosomes requires different procedures and more closely resembles the handling of liposome‐based drugs such as DOX and amphotericin B. More challenging is the need to maintain the protein structure of the exosomes, especially the single‐chain variable fragment, which is crucial for CAR‐T/NK/M‐based therapeutic specificity. Administering CAR‐T/NK/M‐derived exosomes but not CAR‐T/NK/M cells themselves also means that the therapeutic agent cannot reproduce inside the patient's body and has to be manufactured repeatedly if repeat doses are needed. The purity and quality of these tExos need to be examined to ensure consistent efficacy. An alternative strategy is to manufacture exosome‐mimetic nanovesicles that may be easier to scale and produce consistently, but more research is required to ensure that the designed mimetic vesicles can match the efficacy of natural exosomes. Combination therapies, including cell‐based therapy and exosome‐based therapy, are also options. CAR‐T/NK/M‐cell‐derived exosomes or CAR‐T/NK/M‐cell‐derived exosome‐mimetic nanovesicles may be given before CAR‐T/NK/M‐cell therapy, and the tumour response may provide helpful information to the patient and doctor regarding the decision of subsequent and potentially more expensive cell therapy. These exosomes may be manufactured and preserved and may be given on‐demand after cell therapy when a booster treatment is needed.

Therapeutic approaches based on RBC‐Exos have advantages such as their abundance and lack of MHC‐related donor/recipient restrictions. These exosomes may be used for their immunomodulatory effects or as carriers. However, RBC‐Exos can carry RBC antigens and may need to be subjected to all compatibility tests before their use, similar to those for RBC transfusion. In addition, unlike the routine production of leukocyte‐poor packed RBCs, which can have sufficiently low numbers of WBCs and platelets for practical transfusion purposes, it is not clear how pure the RBC‐Exos need to be to achieve the therapeutic goals and whether the removal of WBCs and platelet‐derived exosomes may be necessary, feasible or practical with the available technologies.

**FIGURE 5:**
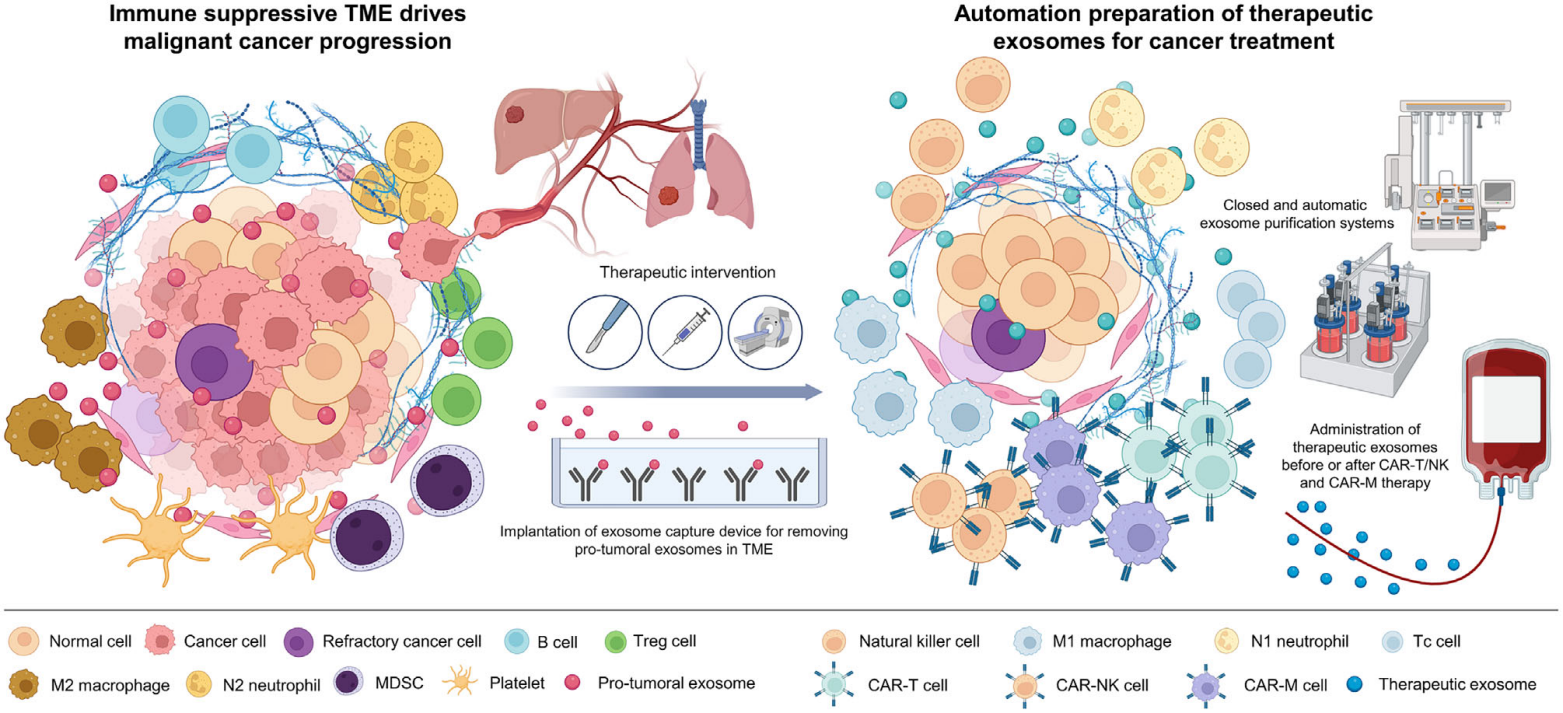
Advancing exosome‐based therapeutics for combating cancer recurrence. Despite significant advancements in cancer treatment, cancer recurrence remains a crucial challenge. After conventional treatments, including surgery, radiation therapy, and targeted therapy, the use of protumoural exosome absorption devices (i.e., depletion of phosphatidylserine‐positive exosomes with T‐cell immunoglobulin and mucin domain‐containing protein 4 [TIM4] capture beads) can simplify tumour microenvironment (TME) complexity. The large‐scale preparation of autologous therapeutic exosomes or the production of exosomes from allogenic universal donors can maintain an immune‐activated TME prior to chimeric antigen receptor T cells (CAR‐T) and chimeric antigen receptor natural killer cells (CAR‐NK) therapies. The figure was created using BioRender. CAR‐M, chimeric antigen receptor macrophages; MDSCs, myeloid‐derived suppressor cells; Tc, cytotoxic T cells; Tregs, regulatory T cells.

Other than using patient‐derived cells, including CAR‐T/NK/M cells, as exosome sources, manufactured exosome‐mimetic nanoparticles, or RBC‐Exos, exosomes from other cellular sources may contain nonidentical MHC molecules compared to the recipient and should be considered allogenic. Whether the administration of exosome‐based therapeutics will induce a sensitisation effect similar to the transfusion of blood products and whether such an effect will prevent repeated administration of the exosomes remain unanswered. Consistent production of sufficient exosomes from CAR‐T/NK/M cells or RBCs may also be challenging and potentially require the combined expertise of bioengineering and blood banking. A bioreactor approach, a multiple donor‐pooling approach, or a single‐donor apheresis approach may be suitable for different scenarios. tExos could be collected from genetically pre‐engineered host cells or purified for downstream physicochemical decoration to facilitate targeted delivery and cancer treatment.^148^ The exosome‐mediated delivery of Cas9 ribonucleoprotein complexes further endows tissue‐specific gene therapy.^149^, ^150^ Experience from exosome‐assisted CAR‐T/NK/M therapy could further facilitate the development of personalised therapy using tissue organoid‐derived exosomes.^151^ Because exosome preparations may carry infectious agents, exosomes derived from autologous or allogenic cells should be manufactured in a closed culture/purification system and then subjected to microbiology testing (Figure 5). Good Manufacturing Practice, Good Laboratory Practice and Good Clinical Practice certificates are essential for the homogeneous scaling of the production of bioactive and tExos. The phenotypical characterisation of concentrated exosomes must be examined according to the Minimal Information for Studies of Extracellular Vesicles 2018 guidelines.^152^

Despite these challenges, exosome‐based therapies remain promising because they differ from molecule‐ and cell‐based therapies. Exosome‐based therapies will find their niche in future cancer treatment by leveraging their improved cell specificity compared to molecule‐based therapies and more straightforward composition and preparation compared with cell‐based therapies.

## CONFLICT OF INTEREST STATEMENT

The authors declare they have no conflicts of interest.

## CONSENT FOR PUBLICATION

All authors have agreed to publish this manuscript.

## Data Availability

Not applicable.
